# The MIPAM trial: a 12-week intervention with motivational interviewing and physical activity monitoring to enhance the daily amount of physical activity in community-dwelling older adults – a study protocol for a randomized controlled trial

**DOI:** 10.1186/s12877-020-01815-1

**Published:** 2020-10-20

**Authors:** Rasmus Tolstrup Larsen, Christoffer Bruun Korfitsen, Carsten Bogh Juhl, Henning Boje Andersen, Jan Christensen, Henning Langberg

**Affiliations:** 1 Department of Public Health, Section of Social Medicine, University of Copenhagen, Copenhagen, Denmark; 2 Musculoskeletal Statistics Unit, The Parker Institute, Bispebjerg and Frederiksberg Hospital, Copenhagen, Denmark; 3 Research Unit of Musculoskeletal Function and Physiotherapy, Institute of Sports Science and Clinical Biomechanics, Faculty of Health Sciences, University of Southern Denmark, Odense, Denmark; 4 Department of Physiotherapy and Occupational Therapy, Copenhagen University Hospital, Gentofte, Denmark; 5 Technical University of Denmark, DTU Management Engineering Inst, Lyngby, Denmark; 6 Department of Occupational- and Physiotherapy, Copenhagen University Hospital, Rigshospitalet, Copenhagen, Denmark; 7 National Centre for Rehabilitation and Palliative Care, University of Southern Denmark and Odense University Hospital, Odense, Denmark; 8 Department of Public Health, Section for Health Services Research, University of Copenhagen, Copenhagen, Denmark

## Abstract

**Background:**

Physical Activity Monitors (PAMs) have been shown to effectively enhance level of physical activity (PA) in older adults. Motivational interviewing is a person-centred model where participants are guided using self-reflection and counselling, and addresses the behavioural and psychological aspects of why people initiate health behaviour change by prompting increases in motivation and self-efficacy. The addition of motivational interviewing to PA interventions may increase the effectiveness of PAMs for older adults.

**Methods:**

This motivational interviewing and PA monitoring trial is designed as an investigator-blinded, two arm parallel group, randomized controlled superiority trial with primary endpoint after 12 weeks of intervention. The intervention group will receive a PAM-based intervention and motivational interviewing and the control group will only receive the PAM-based intervention. The primary outcome is PA, objectively measured as the average daily number of steps throughout the intervention period. Secondary outcome measures include self-reported PA health-related quality of life, loneliness, self-efficacy for exercise, outcome expectancy for exercise, and social relations. The outcomes will be analysed with a linear regression model investigating between-group differences, adjusted for baseline scores. Following the intention to treat principle, multiple imputation will be performed to handle missing values.

**Discussion:**

A moderate effect of daily PA measured using PAMs is expected in this superiority RCT investigating the effect of adding motivational interviewing to a PAM intervention. According to the World Health Organization, walking and cycling are key activities in regular PA and should be promoted. To increase the general public health and lower the burden of inactivity in older adults, cost-beneficial solutions should be investigated further. If this RCT shows that motivational interviewing can enhance the effect of PAM-based interventions, it might be included as an add-on intervention when appropriate. No matter what the results of this study will be, the conclusions will be relevant for clinicians as the dependence on technology is increasing, especially in relation to public health promotion.

**Trial registration:**

NCT03906162, April 1, 2019.

## Background

Twenty-seven percent of older adults in Denmark (65–74 years) and 39–46% of very old adults (age above 75) do not meet the World Health Organization’s (WHO) recommendations for minimum physical activity (PA) [1] and the motivation for increasing PA is low for both age groups [2]. Physical inactivity and low PA levels have a major impact on global public health [3]. Physical inactivity among older adults is associated with disability and premature death and is one of the main barriers to healthy aging [4, 5]. Increased PA levels among older adults, including the ones living with chronic diseases, are associated with longevity benefits and healthy aging no matter the previous level of PA [5, 6].

Overall, PA promoting interventions do seem wo work well among older adults [7, 8] and furthermore, a review of reviews by Olanrewaju et al. found that behavioural and cognitive interventions are effective for increasing short-term PA in older adults [9]. Walking is the preferred form of PA among community-dwelling older adults [10], and participation in walking programs is an effective [9] means of increasing PA levels among this population. In order to maintain long-term participation in PA programs, individualized interventions based on theories of health behaviour change may be required [4, 9]. Social support may be important for increasing PA in older adults as social support and social networks influence health behaviours [11]. Lack of motivation for, or adherence to, exercise in older adults may be due to low self-efficacy or perceived barriers [12–15]. Physical activity monitors (PAMs) used to provide user feedback can facilitate motivational behavioural change and are often used in interventions to increase the average number of daily steps in older adults [16, 17].

However, PAMs might not be adequate or optimal for all older adults, as individualized goal-setting and social support have been reported as important factors in PA interventions [18]. A strategy including PA monitoring, goal setting [18] and Motivational Interviewing (MI) has been shown to promote maintenance of increased PA behaviour 6 months after intervention [19]. MI is a person-centred model where participants are guided using self-reflection and counselling [20]. MI addresses the behavioural and psychological aspects of why people initiate health behaviour changes by prompting increases in motivation and self-efficacy [21, 22]. In Denmark, MI is already well established among municipality health work with older adults or general practitioners’ counselling of patients [23–29]. Furthermore, studies within older adults have reported MI to increase PA levels among patients with heart failure [30] and hip fracture [31]. Finally, older adults have reported the combination of PAMs and MI to be acceptable in a feasibility study aimed to keep people active after a fall management intervention, which to our knowledge is the only study that combines a PAM-based intervention with MI in older adults [32] Thus, MI shows potential for increasing PA levels and seems especially relevant to include and investigate in combination with a PAM-based intervention among Danish community-dwelling older adults [33, 34].

### Objective

The objective of this RCT is to investigate the effect of MI as an add-on intervention to a PAM-based intervention measured by the average daily step count in community-dwelling older adults above the age of 70. It is hypothesized that: 1) MI will enhance the average daily step count among participants, 2) that MI will affect self-reported PA and quality of life, and 3) that self-efficacy and outcome expectancy for exercise will mediate this effect and explain heterogeneity in the results.

### Trial design

The MIPAM trial is designed as an investigator-blinded, two arm parallel group, superiority RCT with primary endpoint after 12 weeks of intervention.

## Methods

This protocol is reported according to the Standard Protocol Items: Recommendations for Interventional Trials (SPIRIT statement) [35].

### Participants, interventions, and outcomes

#### Study setting

This RCT will be conducted nationwide among the community-dwelling older adults in Denmark.

### Eligibility criteria

Participants will be considered eligible for inclusion if they: 1) are retired and community-dwelling, 2) are at least 70 years of age at the time of enrolment, 3) own a smartphone or tablet able to install the *Garmin Connect application,* 4) have an e-mail address and are able to correspond and complete the study survey, and 5) have hearing abilities sufficient to receive a telephone interview.

Participants will not be considered eligible for inclusion, and hence excluded, if: 1) they have cognitive impairment from moderate to severe dementia or Alzheimer’s disease, 2) they are undergoing active chemotherapy or palliative care for cancer, and 3) or have a major mobility impairment preventing them from walking (e.g. from paralysis, amputations, severe arthrosis or arthritis, multiple sclerosis or Parkinson’s disease).

### Interventions

The control group will receive the PAM intervention and the experimental group will receive both the PAM intervention and a telephone-based MI intervention including goal setting for PA.

#### Physical activity monitor intervention

In a recent systematic review including 21 RCTs, PAMs has been shown to effectively enhance the daily number of daily steps in older adults [16, 17]. The PAM intervention consists of a PAM for everyday use in the intervention period and a pamphlet with information about Danish recommendations on PA in aging populations. The PAM will be the hip-worn Garmin Vivofit 3 monitor linked to a pre-specified *Garmin Connect* account. The participants will receive the PAMs and an installation guide, and will be asked to install the *Garmin Connect* application on their smartphone using a pre-specified ID/password in the app. The *Garmin Connect* application (https://connect.garmin.com/) allows participants to track, view and explore their daily step data. It allows for individual goal-setting on PA or other health related variables e.g. weight management, and it also allows the participants to connect with friends or relatives and create challenges with these. The participants will only be asked to install the application and use the automated goal-setting for daily steps, but they will be allowed to explore and use other functions of the application. Participants with installation difficulties will receive support by telephone from the research team. The participants will be asked to wear the monitor for all waking hours, except when bathing, every day for the 12-week intervention period.

#### Experimental intervention

The experimental intervention combines the PAM intervention with a MI-intervention, delivered by MI-trained physiotherapists (PT). During the 12-week intervention period, the participants will receive seven telephone calls. Using an intervention schedule inspired by the work of King et al. to facilitate initiation and maintenance of behaviour change, calls are delivered in the first, second, third, fifth, seventh, ninth and last intervention week [36].

In this person-centred intervention model, participants are guided through self-reflective counselling consistent with the MI approach [20]. They will receive feedback on their PA and health behaviours in relation to the national recommendations. Consistent with the original MI approach [20], this feedback will also highlight the discrepancy between their health goals and their current health behaviours.

The underlying theoretical perspective used to motivate the participants is derived from the Social Cognitive Theory (SCT) and the Transtheoretical Model (TTM) [37–40]. SCT proposes that to promote individuals’ health behaviours, individuals must believe in their ability to carry out the specific behaviour, and they must also believe in its benefit [41, 42]. Self-efficacy and outcome expectations are key constructs and seen as direct predictors of PA behaviours, and they operate through indirect pathways affecting goal setting and the perception of socio-structural factors [37]. TTM was developed by Prochaska and DiClemente and posits that behaviour change follows a series of stages, which will be assessed by the counsellor; 1) precontemplation (individuals are not participating in any PA and have no intention to do so in the future), 2) contemplation (individuals are not participating in any PA but intend to start doing so in the next 6 months), 3) preparation (individuals intend to start participating in regular PA in the next 6 months and are starting to make small changes in their activity behaviour), 4) action (individuals meet defined criteria for PA but have done so for less than 6 months), and 5) maintenance (individuals have met defined criteria for PA for more than 6 months) [38–40]. A number of factors determine movement through the stages, including cognitive and behavioural processes of change, self-efficacy, and outcome expectancies.

Several theoretical constructs from the SCT and the TTM are addressed by the MI intervention. Personal factors and self-efficacy, in this setting for exercise, will be operationalized by coaching with realistic and measurable goal setting. Self-efficacy as a construct will be measured by the self-efficacy for exercise scale (SEE) [43]. Behavioural factors and outcome expectancies will be operationalized through discussion of benefits and barriers to health behavioural change, which should lead to increased perception of benefits and decreased perception of barriers [13]. Further, discussion of problem-solving approach to address behaviours will lead to an improved ability to do so. Outcome-expectancies will be measured by the Outcome-Expectancies for Exercise-2 scale (OEE-2) [44]. In the SCT, social support is an important construct for behavioural change. Environmental factors and social support will be operationalized through identification of supports for maintenance of health behavioural change, and specific goal setting for using supports, which will lead to increase level of support for the participant’s health behavioural change. Participants will be encouraged to use a variety of supports including family and friends, as well as neighbourhood and community supports. In collaboration with local community partners, a community reference guide has been compiled that enables the counsellor to refer participants to specific community resources (e.g., walking groups).

#### Fidelity

**Fig. 1:**
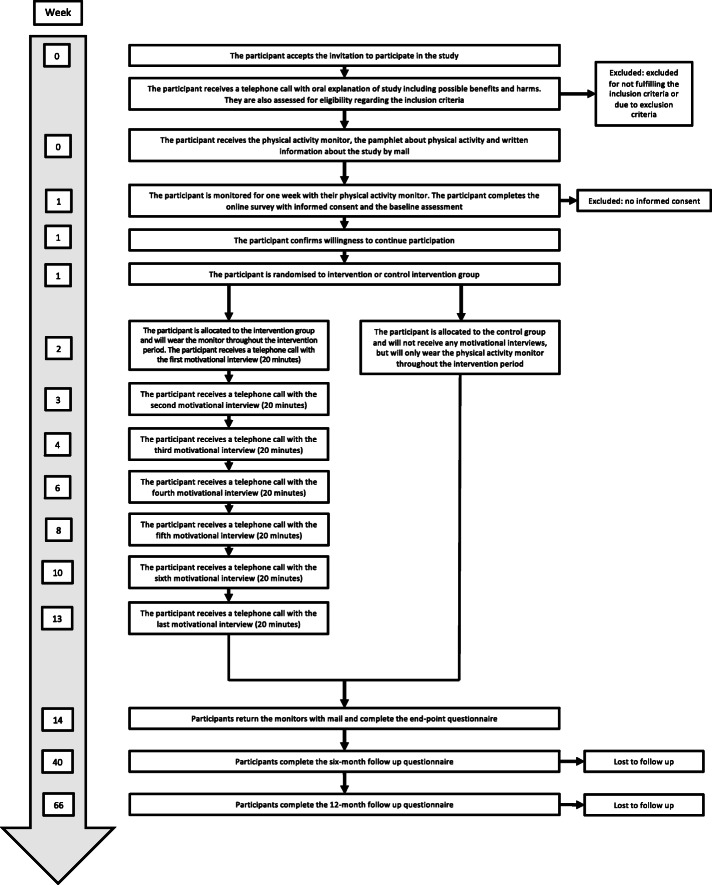
SPIRIT participant timeline

The intervention (and the actual content of the motivational interviews) will be tailored to individual participants, but the number and timing of calls will not be adjusted. The project telephone counsellors are PTs with additional training and education in the MI approach to telephone health behaviour counselling. Training involved a four-day course, with reading materials, discussions with other study investigators and roleplaying exercises. During this study, with participants’ verbal consent, telephone MI sessions will be audiotaped on a regular basis to ensure fidelity of intervention delivery and to provide counsellor feedback. Fidelity monitoring will be conducted by two coders that agree on a global score for each coded MI-session using the Motivational Interviewing Treatment Integrity Scale version 4 (MITI 4) [45]. The MITI 4 consists of four global ratings (Cultivating Change talk, Softening Sustain Talk, Partnership, and Empathy), and 10 individual behaviour counts (Questions, Simple Reflections, Complex Reflections, Persuade with Permission, Giving Information, Affirmations, Emphasize Autonomy, Seeking Collaboration, Persuade and Confront) which are counted in the time frame of the interview [45]. A median global score in each domain of four and a reflection to question ratio of more than one will be considered decent MI proficiency. Call completion, duration of the call, type of MI-intervention and stage of change will be noted after each telephone call (Fig. 1).

## Outcomes

### Primary outcome measure

The average number of steps per day during the first baseline week and the 12-week intervention period is the primary study outcome. The hip-worn Garmin Vivofit 3 tri-axial accelerometer will serve as the PAMs and thus measure the primary outcome. The commercially available Garmin Vivofit 3 has, to the best of our knowledge, only been validated in older adults by our own research group. The Garmin Vivofit 3 was validated with three other monitors and the hip-worn PAMs were found to be superior to wrist-worn PAMs among older adults with and without rollators [46].

### Secondary outcome measures

Secondary outcome measures will include self-reported information from the participants on PA, health-related quality of life, loneliness, self-efficacy for exercise, outcome expectancy for exercise, and social relations. All secondary outcomes will be collected at baseline, at endpoint, and at six- and 12-month follow up.

The baseline self-report questionnaire will be completed before the intervention group receives the first motivational interview and the endpoint questionnaire will be distributed after 12 weeks of intervention and after the last motivational interview.

#### International physical activity questionnaire-short form (IPAQ-SF)

The seven-item IPAQ-SF assesses the amount of moderate to vigorous physical activity (MVPA), VPA, walking time and sedentary time, that has been performed in the past 7 days [47]. The score is categorized into three levels of PA; low, moderate and high [48]. A review of 16 international studies of the measurement properties of the IPAQ-SF assessment demonstrated acceptable reliability (Spearman’s rho: 0,32-0,88) [47] and low to moderate concurrent validity compared to accelerometer with a pooled correlation coefficient of 0.30 (Spearman’s rho range: 0,09-0,38) [49]. The Danish version has previously been used in a Danish population of older adults [50]. MVPA, walking time and sedentary time will be used as outcomes from the IPAQ-SF.

#### Nordic physical activity questionnaire short (NPAQ-short)

The two-item NPAQ-short [51] is a short revised version of the original NPAQ, a survey tool based on telephone interviews designed for the assessment of MVPA. It was developed to monitor compliance with the WHO recommendations on PA [52] and has showed moderate correlation with objectively measured MVPA (Spearman’s rho: 0.33) in a Danish population with an average age of 43 (range: 17–85) [51]. Besides MVPA, the NPAQ-short produces four categories of PA according to the WHO recommendations (inactive, insufficient physically active, sufficient physically active and optimally physically active). MVPA will be used as an outcome from the NPAQ-short.

#### The 5-level EuroQol-5 domain (EQ-5D-5L) quality of life questionnaire

The EQ-5D-5L is a generic Health-Related Quality of Life (HRQoL) measurement tool developed as a non-disease-specific instrument for HRQoL [53, 54]. EQ-5D-5L comprises of five dimensions (mobility, self-care, usual activities, pain/discomfort and anxiety/depression), each of which has five levels (no problems, slight problems, moderate problems, severe problems or unable to), and a visual analogue scale (EQ VAS) [54]. The EQ VAS records the patient’s self-rated health on a vertical visual analogue scale, where the endpoints are labelled ‘The best health you can imagine’ and ‘The worst health you can imagine’. The EQ-5D-5L has shown general feasibility for measuring HRQoL in a population sample of older adults [55]. The test-retest reliability have been evaluated for the EQ-5D-3L index (correlation: 0.67) and the EQ-5D-3L VAS (0.53) [56] but not for the EQ-5D-5L. The EQ-5D-3L has shown fair to moderate convergent validity by correlations with five related domains of the WHO-5 (Spearman’s rho: 0.29–0.61) [57]. The EQ-5D-5L is adapted to Danish [58] but no psychometric evaluation of the Danish version has been published. The EQ VAS score will be used as an outcome from the EQ-5D questionnaire.

#### UCLA loneliness scale

The 20-item UCLA loneliness scale (third version) is a self-report measure of loneliness and social isolation [59]. The scale consists of 11 positive and nine negative items and the total score is calculated as the sum of 20 items (0–60), with a higher score indicating more loneliness. The negative items (one, five, six, nine, 10, 15, 16, 19 and 20) are reversed before the scores are summed (i.e. high score equals less loneliness). The scale is adapted to Danish (translation found in Additional file 2) and has shown high internal consistency (Cronbach’s Alpha: 0.92) and moderate convergent validity with other measures of emotional loneliness (r: 0.69) and social loneliness (r: 0.73). In addition, the scale has showed moderate discriminant validity in relation to self-esteem (r: − 0.58), depression (r: 0,59), extraversion (r: 0.57) and neuroticism (r: 0,58). In a population of older adults, the scale has shown good internal consistency (Cronbach’s Alpha: 0.87) [60]. The total score will be used as an outcome from the UCLA loneliness scale.

#### Self-efficacy for exercise

The nine-item SEE addresses confidence to engage in regular exercise [43], when challenged by known barriers to exercise [61]. The scale was initially developed for sedentary adults living in the community who participated in an outpatient exercise program [62] and was revised to be applicable to older adults [43]. Response categories range from 0 (no confidence) to 10 (very confident) [43]. Item scores are used to calculate a total score (0–90), with higher scores indicating higher confidence, or self-efficacy, related to exercise. The SEE-DK has been translated and adapted to Danish community-dwelling older adults by our research group (translation found in Additional file 4). The average score will be used as an outcome from the SEE-DK.

#### Outcome expectancy for Exercise-2

The 13-item OEE-2 scale was developed from the original 9-item Outcome Expectations for Exercise (OEE) scale that focused on measuring the positive outcome expectations for exercise (POEE). Based on qualitative findings [61, 63], the original OEE was revised to include four items that focused on negative outcome expectations for exercise (NOEE) [44]. It was initially developed for older adults [64, 65]. To complete the OEE2-DK scale the participants are asked, using a Likert scale, to *strongly agree*, *agree*, *neither agree nor disagree*, *disagree*, or *strongly disagree* with each statement of exercising. The POEE and NOEE subscales are scored by calculating the average score on each scale (1–5) and the items three, six, nine and 12 (NOEE subscale) are reversed before the scores are summed [44]. The OEE2-DK has been translated and adapted to Danish community-dwelling older adults by our research group (translation found in Additional file 3). The average score will be used as an outcome from the OEE2-DK.

These secondary outcomes be completed as will be completed as follow-up measures six and 12 months after ending the intervention. They will be conducted as online surveys.

#### Social- and demographic baseline variables

The 42-item Copenhagen Social Relations Questionnaire (CRSQ), will be used to describe participants in terms of structural and social relations. CRSQ was originally developed in Danish in 1999 [66] and measures the structural aspects of social relations, with a focus on frequency and diversity of social contact, and functional aspects with focus on perceived social support. CSRQ has been used in several Danish population-based surveys including in the Copenhagen Aging and Midlife Biobank (CAMB) [67]. In a sample of 38- to 69-year-old adults, the CSRQ showed acceptable face and content validity and good test-retest reliability, with 41% of the items achieving substantial to almost perfect agreement (kappa: 0.65–0.97) and the rest showing moderate agreement (kappa: 0.41–0.60) [68]. The CRSQ will be used to report if the participants are living alone.

Table 1, including socio-demographics of included participants, and Table 2, including PA characteristics, will be used to report relevant baseline information on the participants.

**Table 1:** Socio-demographics of included participants

Characteristics	Overall (n=)	Control group (n=)	Intervention group (n=)	***p***
**Age in years**, mean (95% CI)	–	–	–	⨂
**Male**, n (%)	–	–	–	⊠
**BMI**, mean (95%CI)	–	–	–	⨂
**Education**	–	–	–	⊠
No education, n (%)	–	–	–	
Primary or secondary education, n (%)	–	–	–	
Tertiary education, n (%)	–	–	–	
Master’s degree, n (%)	–	–	–	
**Living alone**, n (%)	–	–	–	⊠
**Long-term illness, injuries or disability more than 6 months,** n (%)	–	–	–	⊠
**Smoking**	–	–	–	
Smokes**,** n (%)	–	–	–	⊠
Quit smoking**,** n (%)	–	–	–	
Never smoked, n (%)	–	–	–	
**Wants to be more physically active**	–	–	–	
Yes, n (%)	–	–	–	⊠
No, n (%)	–	–	–	
Do not know, n (%)	–	–	–	
**Uses a PAM before enrolment**, n (%)	–	–	–	⊠
**Walking aids**				
No walking aids, n (%)	–	–	–	⊠
Cane user, n (%)	–	–	–	
Rollator user, n (%)	–	–	–	
**Reports to be in pain**, n (%)	–	–	–	⊠
**EQ-5D**	–	–	–	
Mobility – reporting problems n (%)	–	–	–	⊠
Self-Care – reporting problems n (%)	–	–	–	⊠
Usual Activity – reporting problems n (%)	–	–	–	⊠
Pain/Discomfort – reporting problems n (%)	–	–	–	⊠
Anxiety/Depression – reporting problems n (%)	–	–	–	⊠
EQ VAS, mean (95%CI)	–	–	–	⨂
**UCLA Loneliness Scale**, mean n (%)	–	–	–	⨂
**OEE average score,** mean (95% CI)	**–**	**–**	**–**	⨂
**SEE average score,** mean (95% CI)	–	–	–	⨂

BMI: Body Mass Index, PAM: Physical Activity Monitor, EQ-5D: EuroQol Research Foundation Five Domains, UCLA: University of California Los Angeles, OEE: Outcome Expectancy for Exercise, SEE: Self Efficacy for Exercise. IPAQ-SF: International Physical Activity Questionnaire-Short Form, NPAQ: Nordic Physical Activity Questionnaire-Short Form, MVPA: Moderate to Vigorous Physical Activity, SD: standard deviation, 95% CI: 95% confidence intervals, IQR: interquartile range, ⨂ Test for between-group difference with unpaired t-test, ⨀ Test for between group difference with Mann-Whitney U test, ⊠ Test for between group difference with Chi^2^ test, *p* values for between group difference ≤.05 are considered significant

**Table 2:** Physical activity characteristics of included participants

Characteristics	Overall (n=)	Control group (n=)	Intervention group (n=)	***p***
**Baseline Physical Activity** Average daily step count**,** mean (95% CI)	–	–	–	⨂
**IPAQ-SF**	–	–	–	
MVPA, mean (95% CI)	–	–	–	⨂
MPA, mean (95% CI)	–	–	–	⨂
VPA, mean (95% CI)	–	–	–	⨂
Walking time, mean (95% CI)	–	–	–	⨂
Sedentary time, mean (95% CI)	–	–	–	⨂
Low activity level, n (%)	–	–	–	
Moderate activity level, n (%)	–	–	–	⊠
High activity level, n (%)	–	–	–	
**NPAQ**	–	–	–	
MVPA, mean (95% CI)	–	–	–	⨂
VPA, mean (95% CI)	–	–	–	⨂
Physically inactive, n (%)	–	–	–	
Insufficient physically active, n (%)	–	–	–	
Sufficient physically active, n (%)	–	–	–	⊠
Optimally physically active, n (%)	–	–	–	

IPAQ-SF: International Physical Activity Questionnaire-Short Form, NPAQ: Nordic Physical Activity Questionnaire-Short Form, MVPA: Moderate to Vigorous Physical Activity, SD: standard deviation, 95% CI: 95% confidence intervals, IQR: interquartile range, ⨂ Test for between-group difference with unpaired t-test, ⨀ Test for between group difference with Mann-Whitney U test, ⊠ Test for between group difference with Chi^2^ test, p values for between group difference ≤.05 are considered significant

### Sample size and power considerations

The estimated number of participants required is 128. This number will be sufficient to show a 0.5 standard deviation difference between groups, equal to a moderate effect size, on the primary outcome (steps per day). The number of participants will yield a power on 80% with a significance level of 0.05. To account for participation attrition, this study will enrol 20% more participants than required, for total of 154 participants divided into two comparison groups.

### Recruitment

We will recruit participants through online advertisements on Facebook and LinkedIn, in non-profit organizations working with older adults (such as activity organizations) and at activity centres and other communities of older adults. Participants eligible for inclusion will receive the information necessary for participation by mail and complete online questionnaires. The participants will only have contact with the research team via phone or e-mail correspondence.

### Assignment of interventions

#### Allocation

##### Sequence generation and allocation concealment mechanism

Participants will be randomly assigned to either the intervention or the control group, with a 1:1 allocation. After completion of the one-week baseline period, eligible participants will be randomized into blocks consisting of a minimum four participants, stratified on sex (M/F) and average daily baseline step count for the baseline period. Randomization of participants will be performed every week, except for weeks with less than four new participants.

Participants will be randomized using the statistical software package STATA. Allocation concealment will be ensured, as the allocation will not be available until the patient has been recruited into the trial, which takes place after the baseline step count measurements have been completed.

One investigator will oversee the randomization. That investigator will receive a list of participant IDs every week and randomize the participants according to the above method. This investigator will not have any role in recruitment or in statistical analyses. The data-analysis-responsible investigator will be blinded for participant allocation. As the primary outcome is objectively measured steps per day, the outcome assessor of the primary outcome can be considered blinded. As the secondary outcome measures are self-reported, the outcome assessor is not blinded.

Due to the nature of the intervention, both participants and staff conducting the motivational interview in the intervention group will not be blinded to allocation. However, they will be strongly encouraged to not disclose the allocation status of the participants with the principal investigator who will conduct the analyses. The group names of the intervention and the control group will be anonymized before the data will be analysed to ensure blinding of the principal investigator.

### Data collection, management, and analysis

#### Data collection methods

This section includes plans for assessment and collection of outcomes.

##### Primary outcome

The primary outcome (average steps per day in the 12-week intervention period) will be extracted from the data management software program Fitrockr. Participants will be asked to synchronize their PAMs and their Garmin Connect application daily, ensuring daily storage of the step counts. Every week, participants who fail to synchronize their PAM will be reminded via e-mail or by telephone. The PAMs have the capacity to store the step counts for 30 days; therefore, no data loss is anticipated, even if the participants fail to synchronize their PAM for longer periods of time. Fitrockr will extract the data from Garmin Connect and make daily step counts available for export through their service. When the participant has completed the 12-week intervention, the daily totals will be extracted as 84 variables (12*7). After the data extraction, the average daily step count will be calculated.

##### Secondary outcomes

All secondary outcomes are participant-reported and administered through the online survey platform SurveyXact. All participants will receive an email with an electronic SurveyXact invitation on the day of randomization. On the last day of intervention (day 84), the participants will receive a similar SurveyXact invitation with the end-point questionnaire. The six- and 12-month follow up assessments will be administered in similar ways as the end-point assessment.

##### Demographic and other baseline items

Non-outcome variables will be included in the baseline questionnaire and thus be self-reported. These variables include: sex (male/female), age in years, height in cm, weight in kg, highest completed education (no education, primary education, secondary education, tertiary education), marital status (married, widow/widower, single, divorced), smoking habits (never smoked, former smokers, smoker), present pain (yes/no), long-term illness or disability from injury (more than 6 months yes/no), felt limited in performing daily activities because of health issues (seriously limited, somewhat limited, not limited), use of walking aids (no walking aids, cane or rollator), use of PAMs before enrolment (yes/no), would like to be more physically active (yes, no, do not know).

Reasons for dropout will be collected from each discontinued participant by the primary investigator after discontinuation.

### Data management

All outcomes data will be collected and stored electronically. No personal data will be exported from Fitrockr or SurveyXact without pseudonymization. Complete anonymization of all data will be performed after the last follow-up period. Data protection agency approval Reference number: 514–0268/18–3000.

Steps per day will be stored each time the participant synchronizes the PAM. The data-handling-responsible program Fitrockr will extract data from the Garmin applications and store these data according to the agreements. When a participant completes the intervention period, their data will be exported from the Fitrockr database and stored securely at the University of Copenhagen server.

### Statistical methods

Distributions of continuous data will be evaluated by inspecting Quantile-Quantile plots of the standardized residuals and histograms with normal distribution curves. Continuous data with normal distributions will be analysed with parametric statistics. Continuous data with non-normal distributions will be analysed as ordinal data with non-parametrical statistics. Categorical data will be presented as frequencies.

The primary outcome, average daily step count, will be analysed with a linear regression model investigating the between-group differences, adjusted for sex (M/F) and baseline daily step count. Following the intention to treat principle, the Gaussian normal regression method will be used to impute missing values (multiple imputation on baseline step count, gender and age).

The same procedure will be used to analyse between group differences on secondary outcomes and all secondary outcomes will be adjusted for baseline score, baseline daily step count and sex (M/F). Harms will be evaluated by calculating the relative risk (RR), separately for serious and non-serious adverse event between the intervention and control group.

In calculating the average daily step count, days with less than 100 steps will be handled as “days of non-wear” and excluded assessing the mean step count.

Table 3 is the outline table for the reporting of end-point values for primary and secondary outcomes.

**Table 3:** End-point values for primary and secondary outcomes, adjusted for sex, baseline scores and baseline daily step count

Characteristics	Control group (n=)	Intervention group (n=)	***P***
**Primary outcome** Average daily step count, mean (95% CI)	–	–	–
**Secondary outcomes**	–	–	–
EQ VAS, mean (95% CI)	–	–	–
UCLA Loneliness Scale, mean n (%)	–	–	–
OEE average score, mean (95% CI)	**–**	**–**	**–**
SEE average score, mean (95% CI)	–	–	–
IPAQ-SF MVPA, mean (95% CI)	–	–	–
IPAQ-SF Walking time, mean (95% CI)	–	–	–
IPAQ-SF Sedentary time, mean (95% CI)	–	–	–
NPAQ MVPA, mean (95% CI)	–	–	–

BMI: Body Mass Index, PAM: Physical Activity Monitor, EQ-5D: EuroQol Research Foundation Five Domains, UCLA: University of California Los Angeles, OEE: Outcome Expectancy for Exercise, SEE: Self Efficacy for Exercise. IPAQ-SF: International Physical Activity Questionnaire-Short Form, NPAQ: Nordic Physical Activity Questionnaire-Short Form, MVPA: Moderate to Vigorous Physical Activity, SD: standard deviation, 95% CI: 95% confidence intervals, IQR: interquartile range, between-group differences calculated from linear regression model, adjusted for baseline scores, sex and baseline daily step count, using imputed values from the Gaussian normal regression method (baseline step count, sex and age). *p* values for between group difference ≤.05 are considered significant

### Monitoring

#### Data monitoring

Not applicable/relevant.

#### Harms

In our study, adverse events will be defined as any unintended negative consequences in a participant without regard to the possibility of a causal relationship with the intervention. Adverse event rates will be measured after the subject has provided consent and enrolled in the study. All adverse events occurring after entry into the study will be recorded. The participants will be asked at the end-point questionnaire if they experienced any adverse events in terms of using the PAMs or trying to enhance their daily amount of PA.

#### Auditing

No auditing has been protocolled.

### Ethics and dissemination

#### Research ethics approval

According to written correspondence with the Danish Ethics Committee in the Capital Region of Denmark, this trial was not subject to the current laws on research ethics in Denmark due to the non-invasive behavioural change intervention. Thus, this study was pre-approved and can be conducted without further approval from the Danish Ethics Committee of the Capital Region of Denmark (Journal-nr.:18004960).

#### Protocol amendments

Any modifications to the protocol which may affect the study procedure, potential benefit to participants, or may affect safety, including changes of study objectives, study design, sample population, sample size, study procedures, or significant administrative aspects will require a formal amendment to the protocol that will be revised and re-uploaded to Clinicaltrials.gov.

#### Consent or assent

Informed consent will be collected electronically via SurveyXact. Prior to agreeing and signing the consent survey, the participant will receive written information about the study by email. If the participant has any questions they may contact the study-responsible researcher. The participant is informed orally and in writing that they can withdraw their consent at any time without affecting current or future treatment in the Danish healthcare system. The translated version of the informed consent material can be found in Additional file 1.

#### Confidentiality

All study-related information and collected data on participants will be stored securely on a server at University of Copenhagen. All extractions from this server will be followed by immediately anonymization of the dataset.

## Discussion

### Implications

We expect a clinically relevant moderate effect on PA from the experimental intervention in this RCT. According to the World Health Organization, walking and cycling are key activities in regular PA and should be promoted among older adults [69–71]. To increase the general public health and lower the burden of inactivity in older adults, the efficacy of cost-beneficial solutions should be investigated further [70]. If this RCT shows that MI can enhance the effect of PAM-based interventions, it might be included as a cost-benefit add-on intervention when appropriate. The conclusions from this study will be relevant for clinicians as the dependence on technology is increasing, especially in relation to public health promotion.

### Methodology

Several recommendations for conducting clinical trials have been published [72, 73] and following the SPIRIT [35] reporting framework does not mean that the trial will be effective. In this section, the most relevant pitfalls in conducting this particular RCT will be discussed.

Unclear hypotheses and multiple objectives often hinder clinical trials as they may confuse readers and lower the applicability [73]. To answer the specific research question about adding MI to PAM-based interventions, this RCT uses a simple design to increase the generalizability of findings outside of the trial context.

If clinically irrelevant outcomes are used, the trail may not reflect the real world concerns of clinicians, which may affect applicability of the trial [74]. Often, surrogate outcomes are used to show an effect if the intervention fails to change the real and clinically relevant outcomes [74]. Thus, HRQoL, outcome-expectancy and self-efficacy of the participants may improve among the intervention group participants and might be considered as positive changes. However, because of the primary outcome of interest, daily PA, is a distinct construct, the secondary outcomes will only be used to explain the effect (or heterogeneity of) in the primary outcome. Daily steps have been shown to be a critical construct as it is highly associated with longevity and health status among older adults [75, 76]. In this study, daily steps will be validly measured by the Garmin Vivofit 3 [46], and thus able to serve as a critical outcome relevant to both clinicians and decision makers.

When selecting eligibility criteria for study participation, researchers should consider whether to strive for a homogeneous or heterogenous sample. For study enrolment purposes, achieving a homogenous sample is more challenging, but may also result in a more precise effect estimate. A heterogenous sample is expected to increase the generalizability of results in exchange for less precise effect estimates and reduced ability to draw conclusions from results [73]. In this RCT, we have chosen eligibility criteria that allow for a quite heterogenic group of study participants, as we expect our results to be most affected by self-efficacy for exercise and outcome expectancy, rather than participant demographics factors [12, 77]. Our study participants may react differently to the behavioural change intervention, but this will most likely reflect the real-life situations where a single approach may not apply equally [4]. In summary, we have chosen generalizability of the results in favour of effect estimate precision.

When choosing the comparator, the control group intervention content, should be relevant and active. Both the PREPARE guideline, CONSORT statement and the SPIRIT checklist suggest building the intervention and control intervention content on a recent published systematic review [35, 72, 78]. Our study group recently published a systematic review that found that PAM-based interventions are effective and that future comparison studies should not use passive control groups to investigate the effect any further [16]. Instead, future studies should investigate the right and relevant questions, such as “does PAM-based interventions work better than …? ” or “can we enhance the effect of a PAM-based intervention by? ” [16]. This RCT stands as a superiority trial investigating if MI should be added to PAM-based interventions among older adults.

Selecting an appropriate study timeline to measure an effect, and in this trial, long-term behavioural change, is critical in trial design [72]. We considered both the practical possibilities and the optimal intervention length and arrived on a 12-week intervention period. If the intervention, and thus exposure to MI is too short the intervention is unlikely to demonstrate positive outcomes. However, a prolonged intervention may hinder implementation in a real-world setting. Among the 21 PAM-based intervention studies included in the previously mentioned systematic review, the median intervention time was also 12 weeks (range 4 to 52). More importantly, the intervention length was not associated with effect size [16]. However, researchers must also include follow-up time to ensure long-term adherence to the health behaviour change, which is ultimately the desired outcome of MI [79]. This RCT will use self-reported measures to investigate long-term adherence to the behaviour change after six- and 12-months after the intervention. Results from these long-term follow up periods will also be published and are expected to be relevant. This is because it is hypothesized that participants who received MI will develop more effective strategies to ensure long-term adherence, compared to the participants who only received the PAM-based intervention.

## Conclusions

PAMs has been shown to effectively enhance PA-levels among older adults and passive comparisons are therefore not encouraged. Future research should investigate whether the effect of PAMs can be enhanced by adding relevant behavioural change content, such as MI, in populations of older adults. This RCT will be conducted according to current best practice guidelines and will help future clinicians and decision makers to decide if MI should be included in PAM-based interventions among older adults.

### Declaration of interests

The content presented within this protocol and the study was produced as part of the project REACH: this project has received funding from the European Union’s Horizon 2020 research and innovation program under grant agreement No. 690425.

## Supplementary information

**Additional file 1.** Informed consent materials.

**Additional file 2.** Danish translation of UCLA.

**Additional file 3.** Danish translation of OEE-2.

**Additional file 4.** Danish translation of SEE.

## Data Availability

The datasets that will be used and analysed during the study will be available from the corresponding author on reasonable request.

## Abbreviations

PAMs: Physical Activity Monitors
PA: Physical Activity
WHO: World Health Organization
MI: Motivational Interviewing
SPIRIT: Standard Protocol Items: Recommendations for Interventional Trials
PT: Physiotherapist
SCT: Social Cognitive Theory
TTM: Transtheoretical Model
SEE: Self-efficacy for Exercise scale
OEE-2: Outcome-Expectancies for Exercise-2 scale
MVPA: Moderate to Vigorous Physical Activity
IPAQ-SF: International Physical Activity Questionnaire-Short Form
NPAQ-short: Nordic Physical Activity Questionnaire short
EQ-5D-5L: 5-level EuroQol-5 Domain
HRQoL: Health-Related Quality of Life
EQ VAS: Visual Analogue Scale
CRSQ: Copenhagen Social Relations Questionnaire
RR: Relative Risk
