# Supplementary material for: The MIPAM trial: a 12-week intervention with motivational interviewing and physical activity monitoring to enhance the daily amount of physical activity in community-dwelling older adults – a study protocol for a randomized controlled trial
Source: BMC Geriatr. 2020 Oct 20;20:412. doi: 10.1186/s12877-020-01815-1 (PMC7576698; doi:10.1186/s12877-020-01815-1)
Supplement: Supplementary file 1 — Additional file 1. Informed consent materials. [file 12877_2020_1815_MOESM1_ESM.docx]

Additional file 1

## Informed consent materials

# *Participant information on participation in a research project*

***Project title****:* *The MIPAM trial: A 12-week intervention with motivational interviewing and physical activity monitoring, to enhance the daily amount of physical activity in community dwelling older adults – a randomized controlled trial*

*This consent form is part of the informed consent process. It is designed to give you an idea of what this research study is about and what will happen to you if you choose to be in the study. The research project is conducted by the primary investigator and PhD fellow Rasmus Tolstrup Larsen, from University of Copenhagen. You must understand what the content and aim of the research project is, before you decide whether you would like to participate in the project. It is important that you do a thorough read of this document.*

*It is possible to receive a telephone call with an oral elaboration of this document. It is also possible to include another person (relative or friend) in the telephone call. If you decide to participate, you have to declare it in an electronic consent form (the link is available in the email you received with this document). Remember that you have the right to consider your decision before you agree to participate. It is completely voluntary to participate in the study and you can by any time chose to withdraw from the study.*

***Aim of the study***

*The aim of this randomized controlled trial is to find new and effective ways to enhance the daily amount of physical activity in older adults. The study will investigate if motivational interviewing as an add-on intervention will enhance the expected effect from physical activity monitors worn daily. All participants will receive a Garmin Vivofit 3 physical activity monitor to wear every day in 13 weeks. Half of the participants will be randomized to receive seven telephone calls with motivational interviewing.*

*We aim to include 154 participants for this project.*

***Trial plan***

*You will receive the physical activity monitor by mail. In the second week you will receive the baseline questionnaire via e-mail. If you are allocated to receive the motivational interviewing, you will receive seven telephone calls (week 2,3,4,6,8,10 and 13).*

*After wearing the physical activity monitor for 13 weeks, you will need to answer the end-point questionnaire and return the physical activity monitor via mail.*

*You will also receive a follow up questionnaire after six and 12 months.*

*As this study only contains a physical activity monitor and motivational interviewing, we do not expect any side effects from the intervention. However, if you suddenly enhance your daily level of physical activity a lot, you may have days where you feel more tired and if you have degenerative changes (e.g. arthritis) you may also have some days where you feel a bit more pain than you are used to.

After the project, we may ask you to participate in a qualitative interview with one of the investigators. This is also completely voluntary.*

***To be eligible for inclusion in this study:***

- *You must be retired and above 70 years of age*
- *You must be able to walk independently without other people assisting you (rollators and canes are allowed)*
- *You are community dwelling*
- *You have a Windows- or Google smartphone, an iPhone or an iPad to install the Garmin Connect application on.*

*Apple: iOS 10.0 or newer. Windows: Windows 10 Mobile version 10586.0 or newer, Windows 10 version 10586.0 or newer.*

- *You have access to an email account.*

***To be eligible for inclusion in this study you cannot:***

- *Have dementia or Alzheimer’s disease*
- *Receive active cancer treatment (e.g. chemotherapy)*
- *Be disabled due to severe diseases or conditions such as Parkinson’s disease, post-stroke paralysis, amputations and others.*

***Financial compensation***

*Your participation in this trial is voluntary and you will not be compensated financially.*

***Personal data management***

*All information about you will be handled according to current Danish data rules and regulations.*

*Personal information such as name, address, email and telephone number will only be stored in closed servers at University of Copenhagen. The Data Protection board approval number for this trial is 514-0268/18-3000.*

*Your data will be anonymized after your participation to use for data analysis in this project and possibly also other research projects investigating other related questions. At any time, you will be able to get your own results from this study.*

*This research project will be published in a scientific peer reviewed journal. Besides the publication, we will disseminate our findings to health professionals, municipalities, at health conferences and to other interested bodies.*

*By reading this document, we hope that you now are adequately informed about the study to make an informed decision on whether you would like to participate. We have also included the standard document “the rights of participants in health research projects”.*

*If you would like to know more about the study before deciding, please contact,*

*Rasmus Tolstrup Larsen
Gothersgade 160, 3.
1123 København K
E-mail:* [*rala@sund.ku.dk*](mailto:rala@sund.ku.dk) *Tlf.: +4542423007*

*Best regards, Rasmus Tolstrup Larsen*

*Videnskabsetisk Komite (projekt nr.: 18004960)
Version 1, 1. april 20*
