# Supplementary figures and images for: The MIPAM trial: a 12-week intervention with motivational interviewing and physical activity monitoring to enhance the daily amount of physical activity in community-dwelling older adults – a study protocol for a randomized controlled trial
Source: BMC Geriatr. 2020 Oct 20;20:412. doi: 10.1186/s12877-020-01815-1 (PMC7576698; doi:10.1186/s12877-020-01815-1)

## Additional file 2

## Danish translation of UCLA


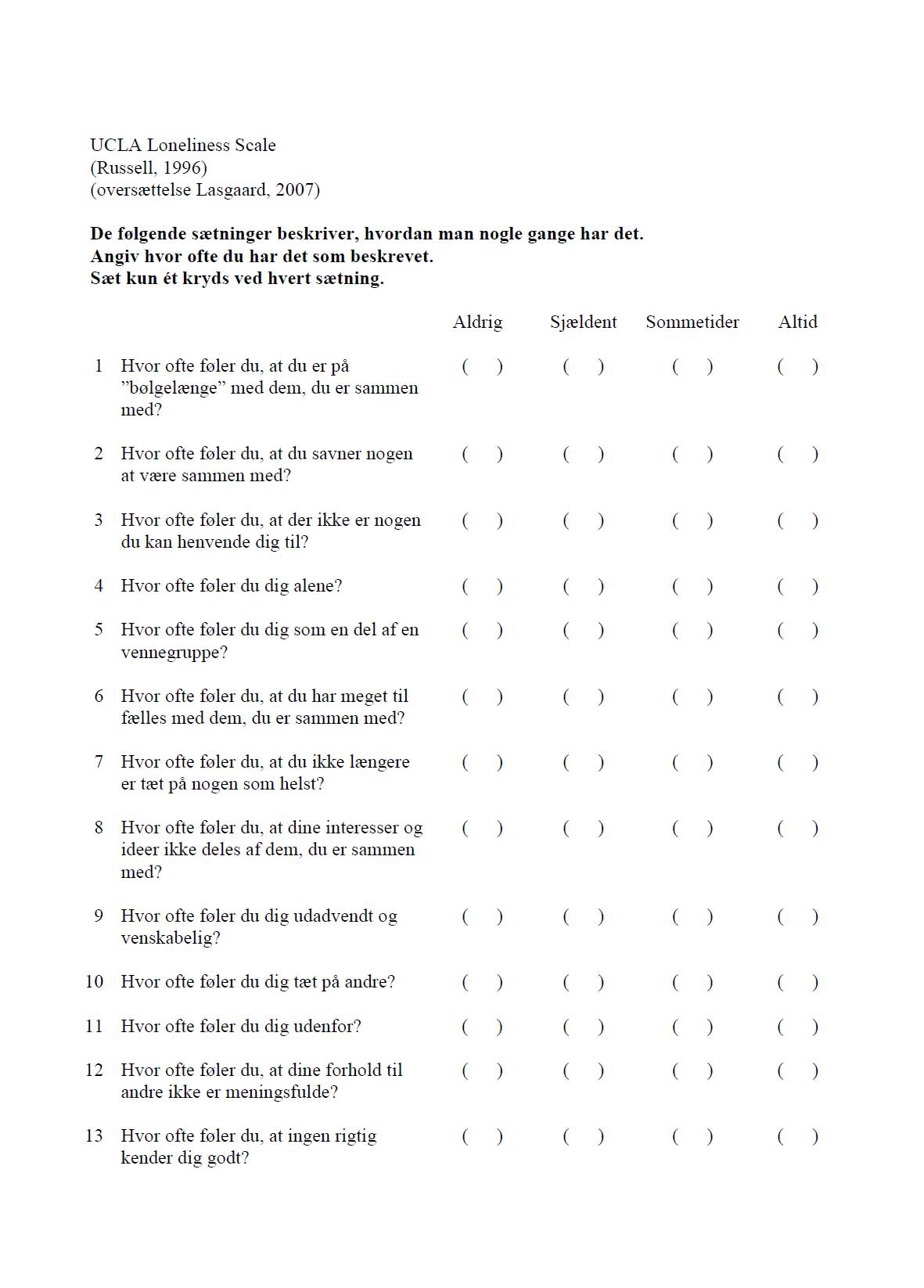

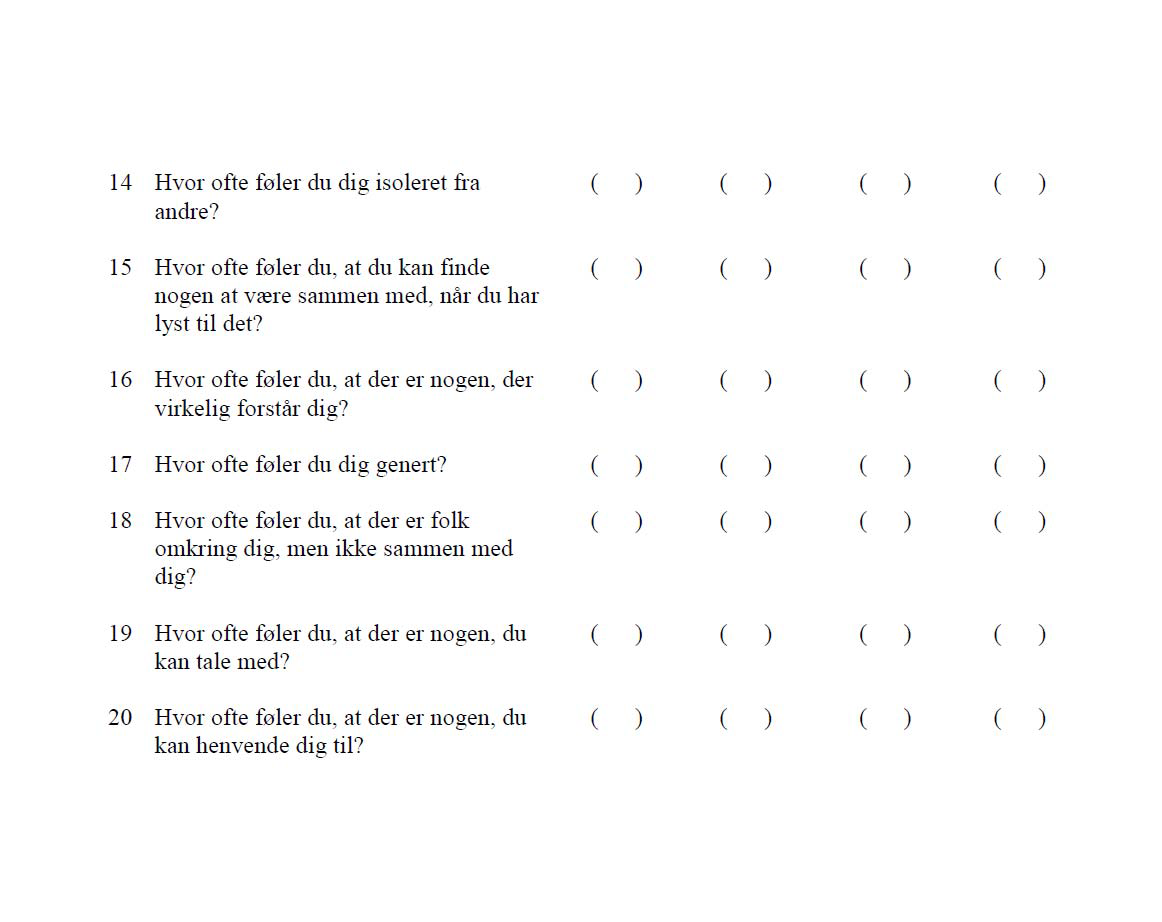

Supplement: Supplementary file 2 — Additional file 2. Danish translation of UCLA. [file 12877_2020_1815_MOESM2_ESM.docx]
