# Supplementary material for: The MIPAM trial: a 12-week intervention with motivational interviewing and physical activity monitoring to enhance the daily amount of physical activity in community-dwelling older adults – a study protocol for a randomized controlled trial
Source: BMC Geriatr. 2020 Oct 20;20:412. doi: 10.1186/s12877-020-01815-1 (PMC7576698; doi:10.1186/s12877-020-01815-1)
Supplement: Supplementary file 3 — Additional file 3. Danish translation of OEE-2. [file 12877_2020_1815_MOESM3_ESM.docx]

## Additional file 3

## Danish translation of OEE-2
